# Commentary: Questioning the HIV-AIDS Hypothesis: 30 Years of Dissent

**DOI:** 10.3389/fpubh.2015.00193

**Published:** 2015-08-07

**Authors:** Alexey Karetnikov

**Affiliations:** ^1^Department of Molecular Genetics, University of Toronto, Toronto, ON, Canada

**Keywords:** HIV, AIDS, antiretroviral therapy, AIDS denialism, pseudoscience, public health

A recent Opinion article by Dr. Goodson (1) expresses pseudoscientific views typical of HIV/AIDS denialism (2–13) and ignores the overwhelming evidence that HIV is a causative agent of AIDS, the evidence accumulated during more than 30 years of research.

## Fulfilling the Koch’s Postulate 1: HIV is Invariably Epidemiologically Associated with AIDS

Dr. Goodson ignores the fact that Koch’s postulates for viruses have been completely fulfilled in the case of HIV (9, 14, 15).

The overwhelming evidence suggests an invariable epidemiological association of HIV with AIDS. AIDS occurs exclusively in HIV-infected people (16). HIV can be detected in all AIDS patients (17). High levels of HIV in the organism predict progression to AIDS (18–23). Many children born to HIV-infected mothers have developed AIDS and died (24). AIDS-related conditions, such as *Pneumocystis* pneumonia and disseminated *Mycobacterium avium* complex disease, have become much more common after the start of the HIV epidemic (25). Death rates are much higher in HIV-seropositive treatment-naïve than in seronegative individuals (26–34).

An HIV-triggered decrease in CD4^+^ T-lymphocyte count is a specific feature of HIV infection, and is extraordinarily rare in the absence of HIV (16, 35–37). The HIV-caused CD4^+^ T-lymphocyte depletion occurs through at least two mechanisms. (1) Direct killing of infected CD4^+^ T-lymphocytes. Dr. Goodson seems unfamiliar with the fact that HIV-1, HIV-2, and other representatives of the genus *Lentivirus* (e.g., Simian immunodeficiency virus), as well as some other retroviruses (e.g., Feline leukemia virus and members of the Avian leukosis virus group), exert a cytopathic effect in infected cells (38). (2) HIV directly kills Th17 CD4^+^ T-lymphocytes in the intestinal submucosa, triggering the damage of the mucosal integrity, translocation of microbial products from the intestine to the blood and chronic immune activation, resulting in further massive loss of CD4^+^ T-lymphocytes (39, 40).

Dr. Goodson claims that recreational drug use, clotting factor VIII, or receptive anal intercourse, but not HIV, are causes of AIDS. However, all of these claims have long ago been rejected by overwhelming scientific evidence (16, 35–37, 41–45).

## Fulfilling the Koch’s Postulate 2: HIV has been Isolated from Patients at all Stages of the Infection

Contrary to Dr. Goodson’s claims, HIV has been isolated *from patients* at all stages of HIV infection, including AIDS, and propagated in cell culture (17, 46–54). Various protocols for HIV-1 isolation (without “contaminants” claimed by Dr. Goodson) have been developed, and each of these protocols can be considered “standard” (55–62). Detailed images of HIV-1 virions, revealing morphology typical of the genus *Lentivirus*, have been obtained using transmission electron microscopy (46–48, 53, 63) and electron cryotomography (64, 65). A combination of immunofluorescent and electron microscopy has allowed visualization of intracellular trafficking of individual HIV-1 particles toward the nucleus of the infected cell (66). The process of cell-to-cell transfer of HIV-1 between T-lymphocytes has been visualized using high-speed three-dimensional video microscopy (67).

## Fulfilling the Koch’s Postulate 3: Accidental HIV Transmission in Humans

Dr. Goodson ignores several tragic cases of accidental HIV transmission to laboratory workers who worked with purified HIV-1, became infected after a needle-stick or mucosal exposure and developed AIDS-like symptoms. HIV has been isolated from their blood, and DNA sequencing confirmed that the HIV variant isolated was identical to the one they were working with (15, 68–70). Other well-documented cases include HIV transmission from a dentist in the USA to several patients (15, 71), and HIV transmission through blood transfusion to 11 children in the USA (72) and 75 children in the former Soviet Union (73).

In addition, the Koch’s postulates for HIV and another lentivirus, Simian immunodeficiency virus, have been fulfilled in experiments with animal models (15, 74).

## HIV Laboratory Testing

Three types of assays are used for HIV detection: (1) ELISA – specificity 98.5–99.9% (75–77), (2) Western blot (77), and (3) PCR – specificity 98.3–100% (78–80). The probability that both ELISA and Western blot would give false-positive results is extremely low (<1/140,000) (77). Thus, contrary to Dr. Goodson’s claims, these tests are highly specific for HIV-1. Since the diagnosis is based on the combination of the three tests (77), HIV testing will produce similar conclusions irrespective of the country.

Dr. Goodson misrepresents the study by Rodriguez et al. (81), which has never stated that PCR “is not sufficiently accurate” (1).

## Antiretroviral Therapy

**Figure 1:**
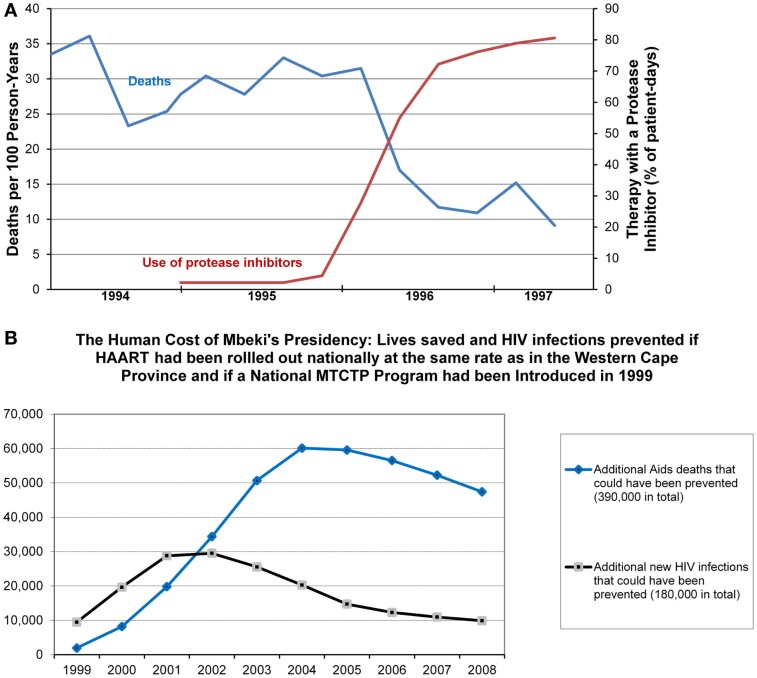
**Contrasting impacts of HIV/AIDS science versus HIV/AIDS denialism on public health**. **(A)** Mortality and frequency of use of protease inhibitor-based combination antiretroviral therapy among HIV-infected patients with fewer than 100 CD4^+^ T-lymphocytes per cubic millimeter, in January 1994–June 1997. Reproduced from Ref. (91), with permission from Massachusetts Medical Society©. **(B)** Estimating the human costs of Mbeki’s AIDS policies implemented with the direct support of HIV/AIDS denialists. Reproduced from Ref. (12), with permission from the Author.

Contrary to Dr. Goodson’s claims, antiretroviral therapy (ART) has profoundly improved the prognosis for HIV-1-infected patients, suppressing their viral load, restoring CD4^+^ T-lymphocyte count, and reducing the risk of developing AIDS or dying (Figure 1A) (82–104). The success of ART has been determined by its high specificity for HIV-1-encoded proteins (105, 106). Along with therapeutic agents for many other diseases, ART does have side effects, but these are far outweighed by its benefits (106). New anti-HIV agents should help to mitigate side effects, overcome drug resistance, and ultimately cure HIV infection, e.g., through excising HIV proviral DNA from the chromosome (107–109).

Dr. Goodson misrepresents the study by the ART Cohort Collaboration, which showed that ART is extremely beneficial for HIV-infected patients, but better clinical outcomes are observed when CD4^+^ T-lymphocyte counts at the start of ART are higher than 200 cells/μl (110). These conclusions have been corroborated by many other studies (111–117) and serve as a background to recommend starting ART early, when the HIV-triggered damage of the immune system is easier to restore (106).

## Detrimental Impact of HIV/AIDS Denialism on Public Health

P. Duesberg, D. Rasnick, and some other HIV/AIDS denialists served on a controversial advisory panel of the South African president Thabo Mbeki. The policy of the South African government over HIV/AIDS during the period 2000–2005 is considered by a majority of scientists to have resulted in the death of at least 330,000 HIV-infected people (Figure 1B) (9, 12, 118). The Opinion article by Dr. Goodson (1) [as well as earlier published or publicly expressed opinions of P. Duesberg, K. Mullis, and other denialists, none of whom has ever worked with HIV/AIDS (2–5, 7–12)] is similarly harmful for public health, as it disseminates dangerous misinformation about HIV/AIDS that can affect prevention decisions made by uninfected people and treatment decisions made by HIV-infected people. Therefore, the following recommendations should be given to public health workers: (1) to learn and disseminate up-to-date knowledge on HIV/AIDS based on the most recent scientific literature, and (2) to be aware of HIV/AIDS denialism and be able to effectively counteract its detrimental impact on public health.

## Conflict of Interest Statement

The author declares that the research was conducted in the absence of any commercial or financial relationships that could be construed as a potential conflict of interest.
